# Analysis of Key Clinical Variables and Radiological Manifestations Associated with the Treatment Response of Patients with Brain Metastases to Stereotactic Radiosurgery

**DOI:** 10.3390/jcm11154529

**Published:** 2022-08-03

**Authors:** Peng Du, Hongyi Chen, Li Shen, Xiao Liu, Jiawei Chen, Xuefan Wu, Tonggang Yu, Daoying Geng

**Affiliations:** 1 Department of Radiology, Huashan Hospital, Fudan University, Shanghai 200040, China; pdu20@fudan.edu.cn; 2 Center for Shanghai Intelligent Imaging for Critical Brain Diseases Engineering and Technology Research, Shanghai 200433, China; 3 Academy for Engineering and Technology, Fudan University, Shanghai 200433, China; hychen20@fudan.edu.cn; 4 Department of Radiology, Jiahui International Hospital, Shanghai 200233, China; li.shen@jiahui.com; 5 School of Computer and Information Technology, Beijing Jiaotong University, Beijing 100044, China; xiaoliu@bjtu.edu.cn; 6 Department of Neurosurgery, Huashan Hospital, Fudan University, Shanghai 200040, China; 20111220062@fudan.edu.cn; 7 Department of Radiology, Shanghai Gamma Hospital, Shanghai 200235, China; kgfztt@163.com (X.W.); ytg521@126.com (T.Y.)

**Keywords:** clinical variables, radiological manifestations, edema index, treatment response, brain metastases, stereotactic radiosurgery

## Abstract

Background: Stereotactic radiosurgery (SRS) is considered a promising treatment for brain metastases (BM) with better healing efficacy, relatively faster treatment time, and lower neurotoxicity, which can achieve local control rates above 70%. Although SRS improves the local control of BM, this may not translate into improvements in survival time. Thus, screening out the key factors influencing the treatment response to SRS, instead of the survival time following SRS, might be of more significance. This may assist doctors when making adjustments to treatment strategies for patients with BM. Methods: This is a retrospective review of 696 patients with BM who were treated with SRS at Huashan Hospital, Fudan University between June 2015 and February 2020. According to the patients’ treatment response to SRS, the patients were divided into an improved group (IG) and a progressive group (PG). The clinical data and magnetic resonance imaging (MRI) performed pre- and post-treatment were collected for the two groups. Five clinical variables (gender, age, Karnofsky performance status (KPS), primary tumor type, and extracranial lesion control) and seven radiological manifestations (location, number, volume, maximum diameter, edema index (EI), diffusion weighted imaging (DWI) sequence signal, and enhanced pattern) were selected and compared. A stepwise regression analysis was performed in order to obtain the best prediction effect of a group of variables and their regression coefficients, and finally to build an SRS treatment response scoring model based on the coefficients. The performance of the model was evaluated by calculating the AUC and performing the Hosmer–Lemeshow test. Results: A total of 323 patients were enrolled in the study based on the inclusion and exclusion criteria, including 209 patients in the IG and 114 patients in the PG. In the Chi-square test and *t*-test analysis, the significant *p* values of KPS, extracranial lesion control, volume, and EI were less than 0.05. Moreover, the cut-off values for volume and EI were 1801.145 mm^3^ and 3.835, respectively. The scoring model that was based on multivariate logistic regression coefficients performed better, achieving AUCs of 0.755 ± 0.062 and 0.780 ± 0.061 for the internal validation and validation cohorts, with *p* values of 0.168 and 0.073 for the Hosmer–Lemeshow test. Conclusions: KPS, extracranial lesion control, tumor volume, and EI had a certain correlation with the treatment response to SRS. Scoring models that are based on these variables can accurately predict the treatment response of patients with BM to SRS, thereby assisting doctors to make an appropriate first treatment strategy for patients with BM to a certain degree.

## 1. Introduction

Brain metastases (BM) are the most common intracranial tumors in adults and about 20–40% of patients with cancer have BM during the course of disease [1]. Due to the combined effect of many factors, the incidence rate of BM is increasing all over the world. BM are terminal manifestations of malignant tumors with poor prognosis, and the main purpose of treatment is to alleviate neurological symptoms and improve the quality of life. Currently, treatment methods for BM mainly include surgery, SRS, whole brain radiotherapy (WBRT), chemotherapy, and targeted therapy [2,3]. Owing to better healing efficacy, relatively faster treatment time, and lower neurotoxicity, SRS has been increasingly applied for patients with a limited number of BM [4,5]. Despite the definite efficacy of SRS for BM, which can achieve local control rates above 70% [6], some patients still suffer from local failure after treatment [7,8].

The main concern of previous studies was the factors influencing the survival of patients with BM after SRS treatment [9,10,11,12,13,14]. These findings were important, but as the Guidelines for Multiple Brain Metastases Radiosurgery [15] reported, although the addition of radiosurgery improves local intracranial disease control, this is of uncertain clinical benefit because, for most patients, survival depends on extracranial disease control. In addition, a multi-institutional retrospective study conducted by the International Radiosurgery Research Foundation revealed that the majority of patients who were treated with salvage SRS following WBRT and a subset of patients who received SRS only succumbed to systemic disease as opposed to neurologically associated death [16]. Furthermore, a multi-institutional prospective observational study of 1194 patients with BM who were treated with SRS indicated that, of the 850 deaths, 92% died of extracranial disease progression rather than intracranial lesions [17]. In other words, although SRS improves the local control of BM, this may not translate into improvements in survival time. Thus, we should pay more attention to the factors associated with a patient’s response to SRS treatment, such as routine clinical variables and radiological manifestations. However, few studies have focused on this.

Therefore, we tried to screen out the key clinical variables and radiological manifestations that are significantly associated with the treatment response of patients with BM to SRS by comparing the relevant clinical data and MRI images of patients with improvement and progression after treatment. This may provide some help for doctors when making an appropriate first treatment strategy for patients with BM. Moreover, radiation necrosis and true progression are dramatically similar in both conventional MRI images and clinical manifestations [18]. In order to neutralize the effect of radiation necrosis, all patients in this study who were identified with progressive disease were subsequently refined with perfusion weighted imaging (PWI) and magnetic resonance spectroscopy (MRS), as determined by a multidisciplinary team of experts in radiotherapy, radiology, neuro-oncology, and pathology.

## 2. Methods

### 2.1. Patient Cohort 

This retrospective study was approved by the institutional review board of Huashan Hospital, Fudan University (KY2021-066) and was conducted in accordance with the Helsinki Declaration. The requirement for written informed consent was waived. The data of patients with BM who received SRS treatment at Huashan Hospital, Fudan University between June 2015 and February 2020 were reviewed. Eligible criteria were as follows: (1) pathologically confirmed primary cancer; (2) no more than four brain metastases confirmed by contrast-enhanced T1-weighted imaging (CE-T1WI) MRI; (3) patients who underwent SRS treatment only; (4) complete acquisition images of pre-treatment and follow-up MRI, including T1-weighted imaging (T1WI), CE-T1WI, T2-weighted imaging (T2WI), and DWI. Patients were excluded if any of the following conditions were met: (1) pathological examination was absent or the diagnosis was combined with another primary cancer (*n* = 37); (2) surgery or WBRT prior to SRS treatment (*n* = 29); (3) other concurrent treatments (*n* = 33); (4) cystic metastases, cranial metastases, and leptomeningeal metastases (*n* = 45); (5) patients judged as having stable disease after treatment (*n* = 178); (6) metallic or motion artifacts on MRI images (*n* = 51). Ultimately, a total of 323 patients were enrolled in the study. Predominant clinical profiles, including gender, age, KPS, primary tumor type, and extracranial lesions control (primary tumor control and extracranial metastasis), were recorded. 

### 2.2. Treatment and Response Assessment

All patients underwent SRS treatment using Leksell Gamma Knife^®^ Perfexion^TM^ (Elekta, Norcross, GA, USA). The median radiosurgery volume was 3.27 cm^3^ and the median margin dose was 17 Gy (range, 15–20 Gy). The margin dose was generally prescribed at an isodose line level of 40–70%, with a median of 50%. The median time between pre-treatment MRI and SRS treatment was 0 days (range, 0–5 days).

Treatment responses were evaluated based on the pre-treatment MRI and the follow-up MRI approximately 60 days after treatment. According to The Response Assessment in Neuro-Oncology Brain Metastases (RANO-BM) criteria [19], patients were classified as having a complete response (CR), a partial response (PR), stable disease (SD), and progressive disease (PD). We defined CR and PR as the IG, and PD as the PG.

### 2.3. MRI Protocol

All MRI examinations were acquired using a 1.5T MRI system (SIGNA Excite HD; GE Healthcare, Milwaukee, WI, USA) with an 8-channel phased-array head coil before and after SRS treatment. MRI sequences included T1WI, CE-T1WI, T2WI, and DWI, and the total acquisition time per patient was approximately 18 min.

### 2.4. Image Analysis

All the MRI images were analyzed in consensus by two experienced neuro-radiologists (with 10 and 15 years of experience in central nervous system radiological diagnosis). Radiological manifestations, including location (frontal lobe, occipital lobe, temporal lobe, parietal lobe, cerebellum, brain stem, and other), number, volume, maximum diameter, EI, DWI sequence signal (hypo-intensity, iso-intensity, and hyper-intensity), and enhanced pattern (homogeneous, heterogeneous, and ring-like patterns), were assessed. The EI was calculated according to the method proposed by Kim et al. [20].

### 2.5. Statistical Analysis

In this study, a total of 12 clinical and radiological variables were evaluated. The independent variables were sex (male = 1, female = 0), age, KPS (>70 = 1, 70 = 0), primary tumor type (numbered in a certain order), extracranial lesion control (primary tumor not controlled with extracranial metastasis = 0, primary tumor controlled or no extracranial metastasis = 1, primary tumor controlled with no extracranial metastasis = 2), tumor location (numbered in a certain order), tumor number, tumor volume, maximum diameter, EI, DWI sequence signal (hyper-intensity = 1, iso-intensity/hypo-intensity = 0), and enhanced pattern (homogeneous = 0, heterogeneous = 1, ring-like = 2). Mainstream software (IBM SPSS statistics 26.0 for windows, IBM Corp, New York, NY, USA) was used for statistical analysis of the clinical variables and radiological manifestations.

First, we performed a univariate analysis by using the chi-square test and *t*-test on the discrete and continuous variables in order to examine the univariate association of the radiological and clinical variables with the SRS treatment response. Statistical significance was set at 0.05 and was used to filter significant variables. The cut-off value for the discrete variable was determined by drawing a ROC curve and by calculating the Youden index. Finally, multivariable logistic regression models were built using the selected variables in order to determine independent predictors for predicting patients’ responses after SRS treatment. Initially, all the variables were included, but the best model was derived using a stepwise logistic regression analysis. The regression coefficient of the best model was used to establish the scoring model. In order to evaluate the discrimination and calibration of the scoring model, the ROC curve was drawn, the AUC was calculated, and the Hosmer–Lemeshow test was performed. 

## 3. Results

### 3.1. Patient Characteristics

A total of 323 patients were included in the study based on the inclusion and exclusion criteria, including 209 patients in the IG and 114 patients in the PG. The patients’ demographic and baseline characteristics are shown in Table 1. Patient age ranged from 45 to 81 years. Primary tumor types included lung cancer, breast cancer, colon cancer, kidney cancer, rectal cancer, ovarian cancer, esophageal cancer, gastric cancer, liver cancer, bladder cancer, cervical cancer, and prostate cancer, etc. Tumors were distributed in frontal, temporal, parietal, occipital, cerebellar, thalamus, brainstem, and basal ganglia regions. Tumor volume ranged from 287.92 mm^3^ to 6780.15 mm^3^, and the average tumor volume was (1921.67 ± 65.10) mm^3^. Maximum diameter of the tumor ranged from 0.53 cm to 4.93 cm, with an average of (2.01 ± 0.06) cm. We divided the generation cohort (generation model and internal validation) and validation cohort for the model according to the proportion of treatment responses. There were 193 cases in the generation model (125 cases from the IG and 68 cases from the PG), 65 cases in the internal validation cohort (42 cases from the IG and 23 cases from the PG), and 65 cases in the validation cohort (42 cases from the IG and 23 cases from the PG).

### 3.2. Patient Outcomes

The results from the univariate analysis are shown in Table 2. After setting *p* < 0.05 as the adoption standard, we concluded that KPS, extracranial lesion control, tumor volume, and EI were significant variables.

**Figure 1:**
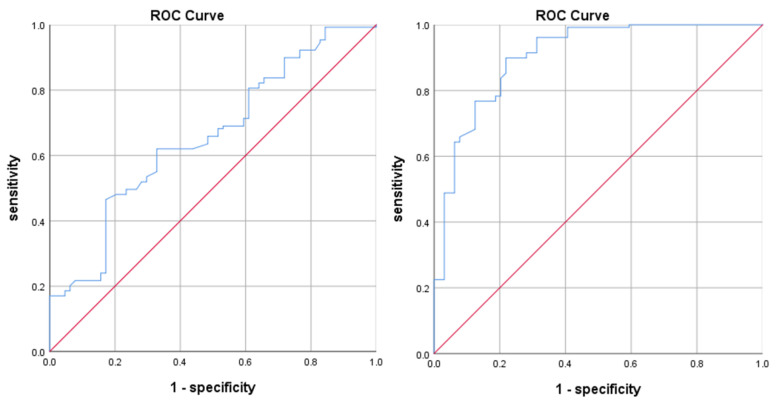
ROC curve for tumor volume (**left**) and EI (**right**).

In order to process the continuous variables, we first drew the ROC curve for two continuous variables (tumor volume and EI) based on the patients’ treatment response, as shown in Figure 1, and calculated the Youden index using sensitivity and specificity. The maximum Youden index for tumor volume and the edema index were 0.347 and 0.706, respectively, and their corresponding cut-off values were 1801.145 and 3.835, respectively. Two continuous variables can be transformed into binary variables by using cut-off values.

We constructed a multivariable logistic regression model based on four variables (tumor volume, EI, KPS, and extracranial lesion control) in order to determine the coefficients of each variable in predicting patients’ treatment responses, and generated a scoring model, as shown in Table 3. Since the coefficients of the regression analysis quantified the magnitude of the effect of the characteristics on treatment response, the score points of the scoring model were obtained by taking the absolute values of the coefficients and rounding them.

**Figure 2:**
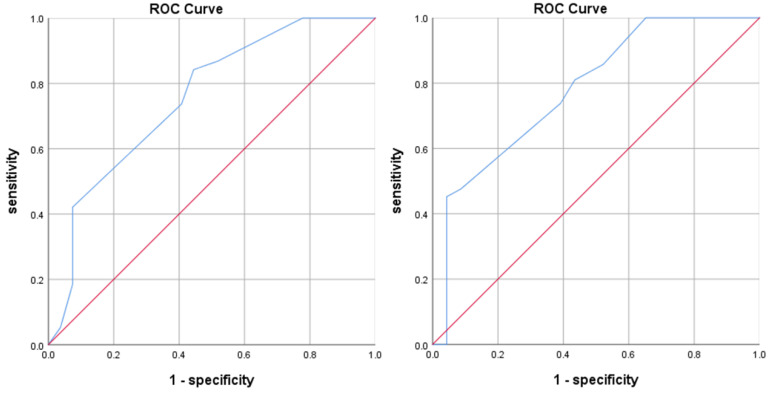
ROC curves for the internal validation (**left**) and validation cohorts (**right**).

Based on the scoring model, we scored the cases in the internal validation cohort and the validation cohort, and used the ROC curve and the Hosmer–Lemeshow goodness of fit test to test the discrimination and calibration of the scoring model. As shown in Figure 2, the AUC value for internal validation was 0.755 ± 0.062, with a 95% CI of 0.634–0.877, and the AUC value for the validation cohort was 0.780 ± 0.061, with a 95% CI of 0.660–0.900. The AUC values were greater than 0.75, indicating that the prediction effect of the model was satisfied.

**Figure 3:**
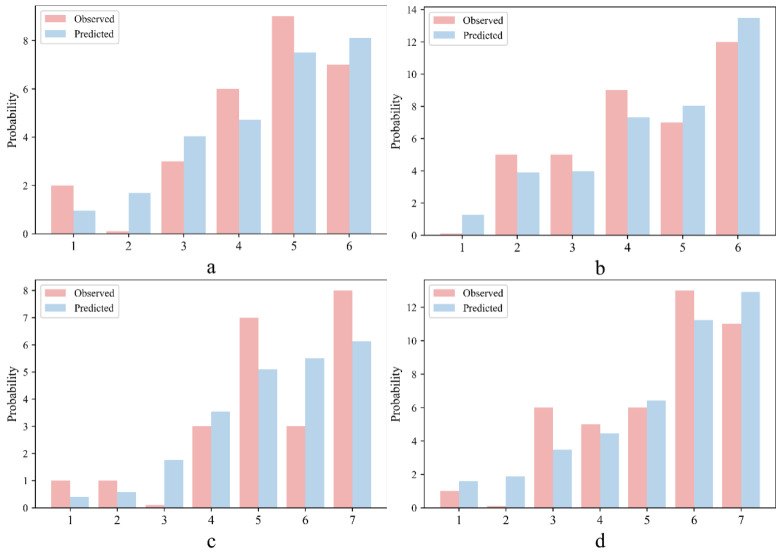
Results of the Hosmer–Lemeshow test for IG (**a**) and PG (**b**) in the internal validation cohort, and IG (**c**) and PG (**d**) in the validation cohort.

In the Hosmer–Lemeshow test, the data for the internal validation and validation cohorts were divided into 6 groups and 7 groups, respectively. The actual observed values and model predicted values corresponding to the treatment response labels of each group are listed in Figure 3. We could intuitively evaluate the performance of the model by comparing the difference between the actual and predicted values. 

The *p* values for the internal validation (χ^2^ = 6.451, *p* = 0.168) and the validation cohorts (χ^2^ = 10.066, *p* = 0.073) were both >0.05, which means that there was no significant difference between the predicted value and the observed value. The above analyses showed that our scoring model has good discrimination and calibration ability.

## 4. Discussion

There are many complex factors affecting the treatment efficacy for BM [21,22], such as the basic situation of patients, the histological type and gene mutation of primary tumors, the control of primary tumors, the patient’s tolerance to chemotherapy, radiotherapy and targeted drugs, the degree of tumor response, the location, number and size of BM, the initial treatment method, and the presence of extracranial metastases, etc. It is quite difficult to cover all the influencing factors in one study. Thus, this study took patients with a KPS ≥ 70, with no more than four BM, who have not undergone surgery or radiotherapy before SRS treatment, and who were not undergoing other treatments concurrently, as the research objects, which reduced the interference of other factors in this research to a certain extent. Moreover, radiation dose, radiation energy, and the mechanisms between radiotherapy and biological individuals are remarkably complicated [23,24]. There is an enormous number of uncontrollable factors and individual biological randomness that affecting the prognosis of patients, so we have not discussed them here.

**Figure 4:**
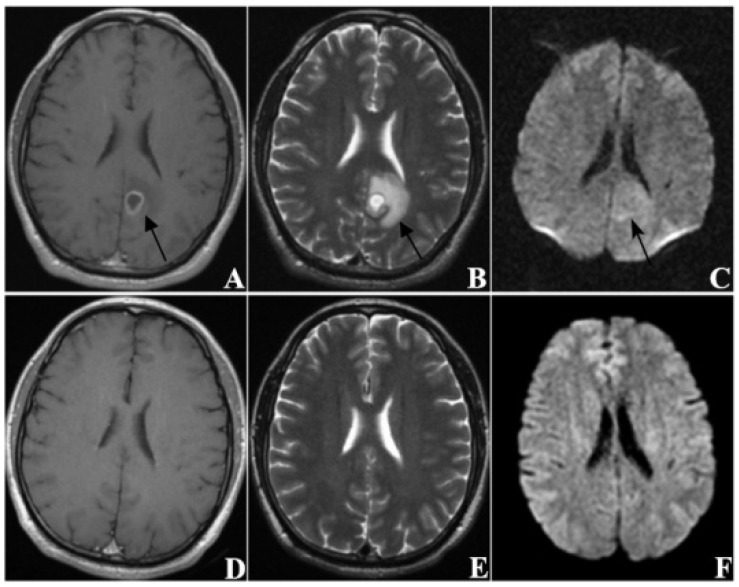
A sixty-three-year-old male patient (KPS = 80) with brain metastases (*n* = 1) from lung cancer. The primary tumor had been completely removed without recurrence and no extracranial metastasis was found. (**A**–**C**) are MRI images taken before SRS treatment, and (**D**–**F**) are MRI images taken at follow-up 63 days after treatment ((**A**,**D**) from CE-T1WI; (**B**,**E**) from T2WI; (**C**,**F**) from DWI). The tumor was located in the left occipital lobe and showed ring-like enhancement. The EI was 3.55. After treatment, the tumor disappeared, thus this patient was classified as CR.

**Figure 5:**
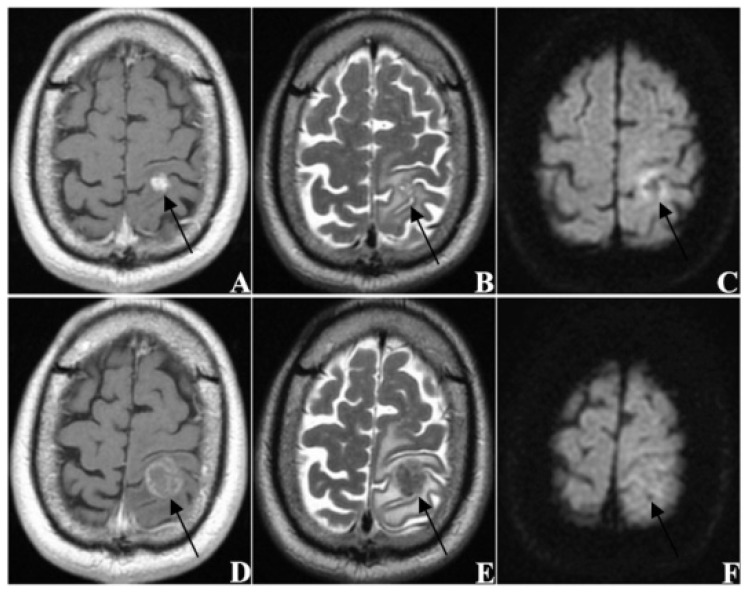
A sixty-year-old male patient (KPS = 70) with brain metastases (*n* = 1) from lung cancer. The primary tumor recurred after surgery with mediastinal lymph node metastasis. (**A**–**C**) re MRI images taken before SRS treatment, and (**D**–**F**) are MRI images taken at follow-up 60 days after treatment ((**A**,**D**) from CE-T1WI; (**B**,**E**) from T2WI; (**C**,**F**) from DWI). The tumor was located in the left frontoparietal junction and showed homogeneous enhancement. The EI was 4.38. After treatment, the tumor was obviously enlarged and the edema was aggravated, thus this patient was classified as PD.

In this study, the MRI images of patients with BM before SRS treatment and at follow-up (approximately 60 days after treatment) were collected in order to evaluate treatment response. In total, there were 209 patients in the IG and 114 patients in the PG. By comparing the differences in clinical variables and radiological manifestations between the two groups, it was found that KPS, extracranial lesions control, volume, and EI significantly correlated with the treatment response of patients with BM to SRS. Figure 4 shows the MRI images of a patient in the improved group pre- and post-treatment, and Figure 5 shows the MRI images of a patient in the progressive group pre- and post-treatment.

KPS is the evaluation standard for the quality of life of patients with cancer and was proposed by Karnofsky from the Eastern Cooperative Oncology Group (ECOG). It was originally used to evaluate whether patients with cancer were able to tolerate chemotherapy [25]. KPS is based on the patient’s condition, normal activity, and self-care. The higher the score, the better the patient’s health will be. At present, KPS is also widely used in the evaluation of patients with cancer before receiving other treatments, and patients with a higher KPS are more able to tolerate the side effects of treatment [26]. Considering the occurrence and development of BM, low KPS before treatment may indicate a poor general condition, immune deficiency, poor control of extracranial lesions (primary tumor and other organ metastases), and that the tumor is more likely to spread and metastasize locally or distantly. It is difficult to control tumors, thereby affecting the efficacy of SRS treatment, which is consistent with the conclusion of this study. A study conducted by Minniti et al. [27] showed that KPS significantly correlated with the prognosis of patients with BM who were treated with SRS. Furthermore, Serizawa et al. [28] believed that systemic disease status and KPS could be used as prognostic factors for patients with BM who were treated with SRS. The findings from a study conducted by Pontoriero et al. [29] showed that a KPS > 70 and stable extracranial lesions were significantly associated with longer survival. Jawahar et al. [30] believed that better control of extracranial lesions after BM was diagnosed was conducive to achieving local control after SRS treatment, and the combination of the two could significantly improve survival rates.

This study indicated that patient age and gender have little correlation with a patient’s treatment response to SRS, which is similar to the findings from research conducted by Petrovich et al. [31]. Although different ages and genders may correspond to different tumor immunities and susceptibility factors, considering that under a reasonable dose of SRS treatment, age and gender do not directly affect the treatment response. In addition, there was no significant correlation between the primary tumor type and the response to SRS treatment in this study, which may be related to the imbalance of primary tumor types in the enrolled cases. In this study, there were 183 patients with lung cancer (56.7%) and 48 patients with breast cancer (14.9%), and the other types of tumors only accounted for 28.4%. Although lung cancer and breast cancer are the most common types of tumors prone to BM [32], it is still necessary to include more types of primary tumors. Furthermore, gene mutations will also affect a patient’s treatment response, to a certain extent. Non-small cell lung cancer, which is the most common type of lung cancer, has many gene mutation types, such as EGFR, ALK, KRAS, BRAF, and ROS1, etc. Different gene mutation types may correspond to different treatments and prognoses [33]. However, this needs to be verified in future research.

Previous studies have shown that BM involving important parts of the brain (brainstem, pituitary, thalamus, and optic nerve) is closely related to the efficacy of SRS treatment. If SRS treatment was given at a conventional dose, it would inevitably adversely affect the important surrounding neuroanatomical structures. Thus, due to the requirement of preserving nerve function, the radiation dose needed to be reduced in order to reduce the local dose, which in turn affected the efficacy of SRS treatment. In this study, there were only 10 patients with BM involving the brain stem, thalamus, and other important parts. These patients accounted for 3.1% of all the included patients, and no correlation between BM location and treatment response was found. Therefore, more patients with BM involving important neuroanatomical structures need to be enrolled in future research.

This study indicated that the number of BM did not significantly correlate with the treatment response to SRS, which was similar to the findings from research conducted by Greto et al. [34]. Bhatnagar et al. [35] found that the correlation between the number of BM and prognosis was not statistically significant. However, a study by Noyama et al. [36] indicated that the number of BM was an independent predictor of intracranial progression-free survival. No more than four BM were included in this study, which may have some impact on the results. Multiple BM may cause greater damage to the brain, resulting in the rapid onset of intracranial hypertension symptoms, which may accelerate progression of the disease and may affect the efficacy of treatment. Thus, whether the number of BM affects the efficacy of SRS treatment and prognosis requires further investigation using a large amount of data.

The findings from a study conducted by Wang et al. [37] suggested that tumor volume was a significant predictor of local control for BM treated with SRS. Yamamoto et al. [17] believed that, for patients with BM who were treated with SRS, an increase in tumor volume was clearly an adverse factor for prolonging survival, whereas factors related to tumor size did not significantly affect the efficacy of SRS treatment and survival. In addition, a study by Baschnagel et al. [38] indicated that tumor volume was a strong independent predictor of local control, distant brain failure, and overall survival in patients with BM who were treated with SRS. In this study, the tumor volume of patients in the IG was significantly smaller than that of patients in the PG. There was no statistically significant difference in the maximum tumor diameter between the two groups. These findings are basically consistent with the results of previous studies. These findings may be related to the radiation dose of SRS treatment, which is characterized by a sharp decrease with off-axis distance. However, the degree of reduction also varies with the volume of the lesion, and the prescribed dose obtained by the tumor is negatively correlated with tumor volume [39].

DWI reflects the Brownian motion of water molecules, which is one of the most important MRI sequences, and hyper-intensity indicates that the motion of water molecules is limited [40]. In this study, almost all the BM before SRS treatment showed hyper-intensity on DWI sequence, which may relate to the growth pattern of highly infiltrating tumor cells. After treatment, most tumors showed iso-intensity or hypo-intensity on DWI sequences, regardless of whether they were in the IG or PG, which may relate to tumor cell necrosis caused by radiation [41]. Therefore, there was no statistically significant difference in DWI sequence signals between the IG and PG. In addition, enhanced patterns of BM are related to a variety of factors, such as the degree of blood–brain barrier damage, angiogenesis, tumor blood supply, and the proportion of solid components. No significant difference in enhanced pattern was observed between the IG and PG. EI has only previously been used in clinical studies investigating meningioma [20], and has not been used in the assessment of SRS treatment efficacy for BM. The greater the EI, the more serious the edema will be. Peritumoral edema in BM is mainly located in the white matter of the brain, which is an indirect sign of the tumor, belonging to vascular edema with complicated causes. In this study, the smaller the EI, the better local control would be after SRS treatment. Reasons for this are complicated and may be related to the size of the tumor and its location, or the pathological type of the primary tumor, which requires validation in future research.

The initial treatment for BM patients relies substantially on local therapy (radiotherapy or neurosurgery) [3], whereas neurosurgery for BM has traditionally been recommended only for patients with a limited number of intracranial lesions [42]. Previous research has reported that potentially clinically informative alterations in the brain metastases that were not detected in the matched primary tumor sample were found in 53% of BM cases [43]. Therefore, for patients with a limited number of BM, once they are considered as being at possible risk of progressive disease after SRS, neurosurgery should be primarily recommended. Removing symptomatic masses through surgical resection may improve the neurological and systemic status of patients. Meanwhile, tumor markers and gene mutations elucidated from surgical pathological specimens can often guide the selection of targeted therapeutic agents for the postoperative systemic treatment of BM patients [2]. Consequently, patients with no more than four BM were included in this study. This is the patient group that is most likely to benefit from initial local treatment. If patients are at risk of progressive disease, surgical resection or surgical resection combined with whole-brain radiotherapy is preferable.

However, our study still has some inevitable limitations. First, this was a retrospective study and not a randomized trial, lending to its inherent limitations. Secondly, treatment responses were evaluated based on the pre-treatment MRI and the follow-up MRI approximately 60 days after treatment. Although it met the evaluation requirements of the RANO-BM criteria, it may only represent the short-term treatment response, and follow-up and long-term efficacy analyses are still needed. Thirdly, the gene detection information of the primary tumors was incomplete. This needs to be added in future research. Moreover, multi-centered, prospective, and randomized controlled clinical research on the treatment response of patients with BM to SRS is required.

## 5. Conclusions

Treatments for BM are changing rapidly, requiring doctors to formulate individualized treatment strategies based on multidisciplinary theories, such as neuro-oncology, radiology, radiotherapy, and pathology, in order to achieve the best treatment efficacy. Valuable clinical variables and radiological manifestations may play an indispensable role in first treatment strategy making. This study indicates that KPS, extracranial lesion control, tumor volume, and the EI for BM patients correlates with the patients’ treatment response to SRS. These findings may assist doctors when making appropriate first treatment strategies for patients with BM.

## Figures and Tables

**Table 1:** Summary of patient characteristics.

Variable	No. of Patients (%)
sex	
male	158 (48.9)
female	165 (51.1)
KPS	
KPS > 70	185 (57.3)
KPS = 70	138 (42.7)
extracranial lesion control	
no control and extracranial metastasis	81 (25.1)
control or no extracranial metastasis	113 (35.0)
control and no extracranial metastasis	129 (39.9)
number of tumors	
1	223 (69.0)
2	71 (22.0)
3	21 (6.5)
4	8 (2.5)
DWI sequence signal	
hyper-intensity	278 (86.1)
iso-intensity or hypo-intensity	45 (13.9)
enhanced pattern	
homogeneous	113 (35.0)
heterogeneous	105 (32.5)
ring-like	105 (32.5)

**Table 2:** Univariate analysis of patient characteristics.

Variable	*p* Value
sex	0.971
age	0.523
KPS	3.620 × 10^−18^
primary tumor type	0.997
extracranial lesion control	7.860 × 10^−20^
tumor location	0.926
tumor number	0.925
tumor volume	1.957× 10^−6^
tumor maximum diameter	0.976
EI	2.928 × 10^−22^
DWI sequence signal	0.127
enhanced pattern	0.998

**Table 3:** Scoring model and results of the regression analysis.

Variables	Score Points	Coefficient	*p* Value
volume > 1801.145 mm^3^	1	1.077	1.957 × 10^−6^
EI > 3.835	2	−2.007	2.928 × 10^−22^
KPS > 70	3	−2.614	3.620 × 10^−18^
extracranial lesion control			
control and no extracranial metastasis	5	−5.475	7.860 × 10^−20^
control or no extracranial metastasis	3	−2.647	7.860 × 10^−20^

## Data Availability

The data presented in this study are available on request from the corresponding author. In order to protect patient privacy, the data are not publicly available.
